# Drug‐Resistant Sinus Tachycardia as a Dose‐Dependent Side Effect of Spironolactone: A Case Report and Review of the Literature

**DOI:** 10.1002/ccr3.71875

**Published:** 2026-02-08

**Authors:** Zahra Abdi, Maryam Chenaghlou, Aysa Rezabakhsh, Laya Hooshmand Gharabagh

**Affiliations:** ^1^ Cardiovascular Research Center Tabriz University of Medical Sciences Tabriz Iran; ^2^ Student Research Committee Tabriz University of Medical Sciences Tabriz Iran; ^3^ Patient Safety Research Center, Clinical Research Institute Urmia University of Medical Sciences Urmia Iran; ^4^ Department of Internal Medicine, School of Medicine Imam Khomeini Hospital, Urmia University of Medical Sciences Urmia Iran

**Keywords:** adverse event, mineralocorticoid receptor antagonist, polycystic ovarian syndrome, reflex tachycardia, spironolactone

## Abstract

Spironolactone is widely used for polycystic ovarian syndrome due to its anti‐androgenic properties. However, rare cardiovascular side effects may occur even in normotensive patients. We report a case of severe palpitations and drug‐resistant sinus tachycardia following high‐dose therapy, emphasizing the need for cautious titration and cardiac monitoring.

## 1 Introduction

Spironolactone is mainly a potassium‐sparing diuretic that acts by blocking aldosterone receptors in the distal renal tubules and collecting ducts, resulting in potassium retention and the excretion of sodium and water [1]. As a result, it may cause hypovolemia and a subsequent drop in blood pressure. Due to its affinity for androgen receptors, spironolactone is also widely used alone or in combination with contraceptive medications to treat PCOS in women of reproductive age, a condition often associated with elevated androgen levels [2, 3].

Hypovolemia‐induced hypotension is detected by arterial baroreceptors, which trigger a reflex increase in sympathetic activity and a decrease in parasympathetic tone. This autonomic response causes vasoconstriction, increased cardiac contractility, and tachycardia, factors that may predispose patients to sinus tachycardia (ST) [4, 5]. ST is a type of arrhythmia resulting in a rapid ventricular rate exceeding 100 beats per minute (bpm) [6].

While spironolactone is best known for its potassium‐sparing diuretic action and inhibition of mineralocorticoid receptor antagonists (MRA), several pathways have been suggested to account for its uncommon association with tachycardia or heightened sympathetic activity. Factors such as hyperkalemia, volume depletion, drug interactions, higher dosing regimens (> 50–100 mg/day), and pre‐existing cardiac or renal disease may contribute to this response. By reducing aldosterone‐mediated sodium retention and vascular tone, spironolactone can lower intravascular volume, and in susceptible individuals this may provoke baroreceptor‐driven increases in heart rate (HR). Under these circumstances, symptoms such as palpitations, marked sinus tachycardia, or infrequent supraventricular arrhythmias may occur, particularly in patients prone to volume fluctuations, hormonal shifts, or comorbid conditions. Based on the literature review, there is little evidence to support an association between spironolactone and tachycardia or reflex tachyarrhythmias. Reported side effects of spironolactone in published studies mainly include hyperkalemia, low blood pressure, menstrual irregularities, and gastrointestinal disorders; while, reflex tachycardia is not typically identified as a cardiovascular adverse effect. This case report presents a young normotensive female patient with PCOS who developed drug‐resistant reflex tachycardia in response to high‐undivided dose spironolactone. This case draws clinicians' attention to the adverse effects of a commonly prescribed medication, even in patients without underlying cardiovascular diseases, and reminds them to monitor patients who are prescribed spironolactone, especially at higher doses.

## 2 Case History

A 37‐year‐old woman with a 17‐year history of PCOS (with symptoms of oily forehead skin, hair loss in an androgenic pattern, and minor menstrual cycle irregularities) and no history of hypertension or diabetes. The past laboratory results indicated normal thyroid function markers, while serum testosterone and dehydroepiandrosterone sulfate (DHEA‐S) levels were elevated compared to the average but remained within the established reference ranges (1.73 pg/dL and 309.5 μg/dL, respectively). As the initial treatment, she was prescribed spironolactone 25 mg three times daily along with cyproterone acetate, under the care of a dermatologist. After the recurrence of her symptoms every 6–12 months, she was prescribed multiple cycles of combined oral contraceptives, such as drospirenone‐containing agents like Yaz and Yasmin, under the supervision of her gynecologist and dermatologist. Since she preferred to stop contraceptive medications, her endocrinologist prescribed a high‐dose spironolactone monotherapy (100 mg daily).

## 3 Differential Diagnosis, Investigations, and Treatment

**FIGURE 1:**
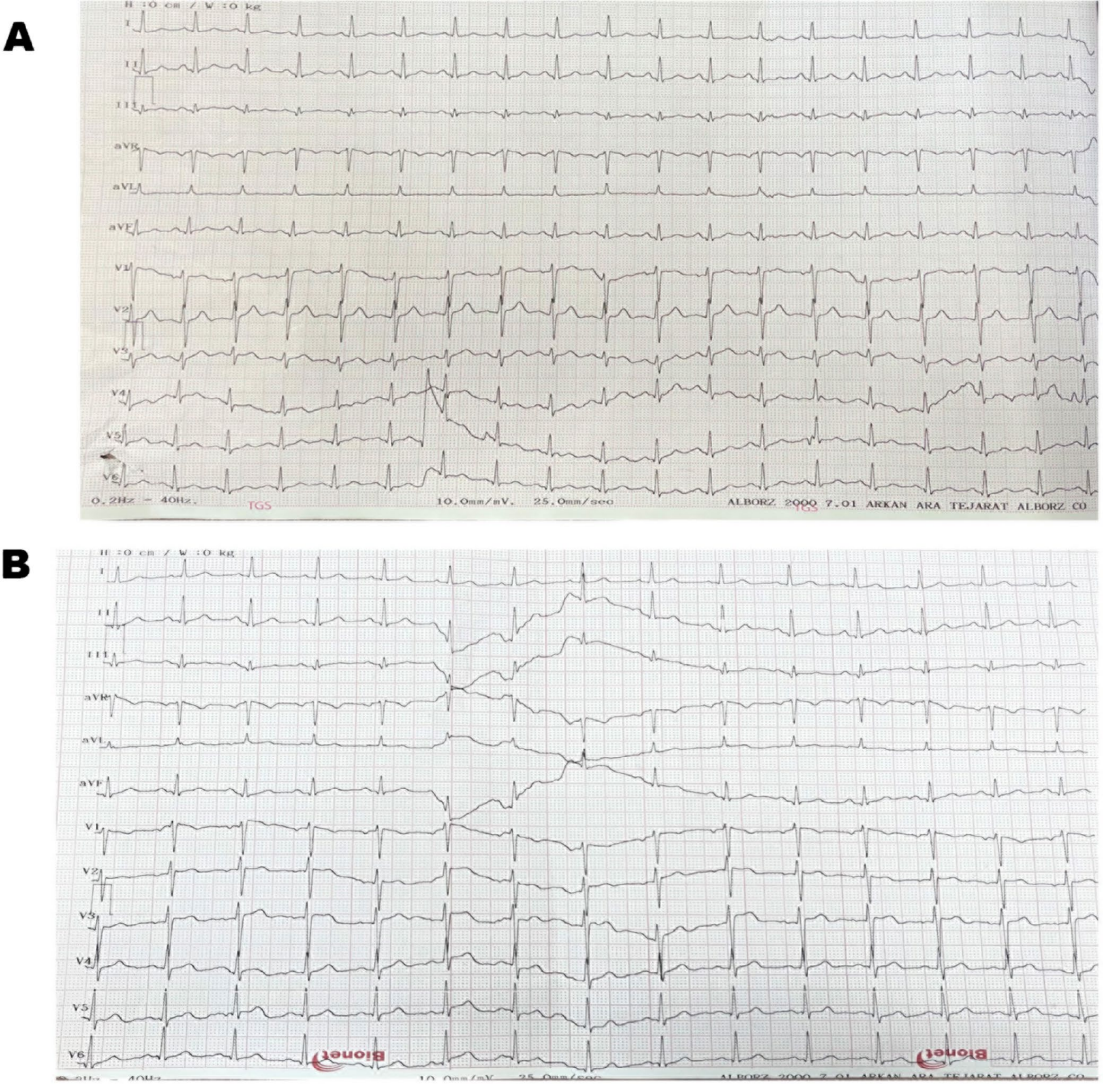
The tracing showed a sinus rhythm with a normal axis. (A) the heart rate of 110 bpm was consistent with reflex tachycardia before dose correction. (B) This part represents the normal sinus rhythm after dose division. All intervals, including PR, QRS, and QT were within normal limits, and no significant ST‐T abnormalities were noted.

In the initial month of therapy, the patient complained of marked palpitations and sought emergency cardiac evaluation for ECG monitoring, while blood pressure remained within normal limits (Figure 1). Despite a normal ECG, a 24‐h Holter monitor was ordered to rule out any serious supraventricular or ventricular tachycardia (VT); however, abnormal HR problems or arrhythmias were not found. In addition, either the thyroid biomarkers or serum electrolyte panels were normal (Table 1). She was given propranolol 20 mg followed by chlordiazepoxide 5 mg for symptomatic management of presumed anxiety‐induced tachycardia; nonetheless, there was no noticeable change in the palpitations.

**TABLE 1:** The assessment of laboratory parameters.

Parameters	Results	Unit	Ref range
ESR 1st hour	22	mm/h	≤ 20
ESR 2st hour	38.0	mm/h	
PT	12.8	Sec	13.5 ± 2
Pt‐activity	100	%	70–100
INR	1.0	—	1.0 ± 0.2
Platelet	286	1000/μL	130–450
Calcium	10.2	mg dL	8.5–10.5
Phosphor	3.9	mg dL	2.5–4.5
Na	137	mEq/dL	135–148
K	4.4	mEq/dL	3.5–5.5
LDH	310	U/L	< 480
TSH	3.75	micIU/mL	0.35–4.94
T4	8.19	μg/dL	4.87–11.72
T3	89.75	ng/dL	35–193

Abbreviations: DHEAS, dehydroepiandrosterone sulfate; ESR, erythrocyte sedimentation rate; INR, international normalized ratio; LDH, lactate dehydrogenase; PT, prothrombin time; TSH, thyroid stimulating hormone.

## 4 Outcomes

After reassessment, her endocrinologist reduced the spironolactone dosage to 50 mg per day. The patient's symptoms resolved entirely within 24–48 h. The dosage was subsequently adjusted to 50 mg twice daily, which was well‐tolerated over the following months without any return of symptoms. The body's compensatory response to the decrease in blood pressure presented as reflex tachycardia, which was unresponsive to the first symptomatic interventions.

## 5 Discussion

The presented case highlights the importance of personalized cardiovascular risk evaluation before the administration of spironolactone, particularly at higher doses. Spironolactone, a medication commonly prescribed in PCOS and heart failure settings, via its diuretic effect, may induce volume depletion and a resultant decrease in blood pressure. This hypotensive state stimulates the baroreceptor reflex, resulting in increased sympathetic tone and decreased parasympathetic activity. The consequent increase in myocardial excitability and HR may promote individuals prone to arrhythmias, including VT. Although this mechanism is theoretically feasible, reports of such effects in young, normotensive individuals without structural cardiac disease are rare. Women with PCOS possess an elevated risk of cardiovascular problems due to metabolic abnormalities such as hyperandrogenism, dyslipidemia, endothelial dysfunction, and increased arterial stiffness [7]. Spironolactone, primarily prescribed for its anti‐androgenic effects, also exhibits cardioprotective properties, enhancing lipid profiles, increasing vascular compliance, and mitigating aldosterone‐induced endothelial injury. These outcomes, which are mostly associated with the inhibition of androgen and mineralocorticoid receptors, indicate its extensive therapeutic applications [3, 8, 9].

Noteworthy, research has shown that spironolactone is effective in individuals with hypertension and diabetes, as it lowers blood pressure, urinary albumin excretion, and markers of inflammation and fibrosis, suggesting its extensive cardiometabolic advantages [10]. Additionally, in patients with HF, both preserved and reduced ejection fraction, MRAs such as spironolactone have demonstrated mortality benefits and a reduction in VT burden, primarily through anti‐fibrotic and remodeling effects. However, comparative studies suggest that eplerenone, another member of MRAs, may offer superior protection against VT recurrence and improved survival, as it acts more selectively than spironolactone [11, 12, 13, 14, 15]. It is worth mentioning that in post‐myocardial infarction patients without apparent HF, routine use of spironolactone has not been associated with significant improvements in cardiovascular outcomes [16].

Besides, the potential anti‐arrhythmic effect of spironolactone in patients with implantable cardioverter‐defibrillators (ICDs) has also been investigated in several studies with conflicting results. In a retrospective multi‐center study of patients with left ventricular systolic dysfunction and ICDs (LVEF ≤ 30%), the addition of spironolactone was associated with a significant decrease in the monthly frequency of shocks, sustained, and non‐sustained VT episodes. However, it did not significantly affect the incidence of ventricular fibrillation (VF) [17].

In this line, spironolactone therapy has been linked to a decrease in the rate of VT episodes in patients with cardiomyopathy. This benefit is also believed to arise from its potent anti‐fibrotic characteristics. In other words, by suppressing fibrosis, spironolactone not only decreases the burden of VT but also slows its rate (i.e., longer cycle length), resulting in episodes that are better tolerated clinically and more likely to be terminated by anti‐tachycardia pacing instead of painful ICD shocks [18]. These findings emphasize that although spironolactone may help reduce ICD therapy in certain high‐risk HF populations, its advantages are likely dependent on patient selection and underlying cardiac function.

Arrhythmogenic cardiomyopathy (ACM) is a genetic disorder associated with a high risk of ventricular arrhythmias caused by abnormal repolarization and calcium regulation in cardiomyocytes [19]. In an experimental model of human‐induced pluripotent stem cell‐derived cardiomyocytes, spironolactone and its metabolite, canrenoic acid, also showed direct anti‐arrhythmic effects, including normalization of action potential duration by suppressing overactive potassium flows and correcting abnormal intracellular calcium dynamics [20]. Notably, these effects were independent of mineralocorticoid receptor blockade, indicating a distinct electrophysiological role for spironolactone in ACM [21].

Conversely, the SPIRIT trial, a randomized, double‐blind study in patients with ICDs who were not candidates for spironolactone according to HF guidelines, failed to show any significant difference between spironolactone and placebo regarding the delay of the first recurrence of VT/VF or reducing overall VT/VF events requiring ICD therapy [22].

On the other hand, in hemodialysis patients, spironolactone has demonstrated both beneficial and concerning effects on cardiac electrophysiology. In a randomized crossover trial, spironolactone increased HR variability, a marker of improved autonomic balance, which reflects reduced sympathetic tone due to aldosterone inhibition at the central nervous system level. However, it also paradoxically increased premature ventricular complexes, possibly because of partial agonist activity at the mineralocorticoid receptor under certain intracellular conditions, raising the possibility of a pro‐arrhythmic effect [23].

A second trial extended these concerns, showing a higher frequency of bradycardia and conduction blocks among patients administered spironolactone, particularly at a dose of 50 mg. Mechanisms involved a slight increase in serum potassium, which was quantitatively greater in the spironolactone group and is a known factor for slowed cardiac conduction [24]. In Table 2, previous studies regarding MRAs' impacts on VT in various subsets of patients have been summarized.

**TABLE 2:** Effects of spironolactone on VT.

No.	Study	Medications, dosage	Disease	Sample size	Adverse event/Primary aim	Main outcome/conclusion
1	[11]	Spironolactone vs Eplerenone (dose not reported)	HF	202	Comparison of the effects of SP and Ep on all‐cause mortality and VT recurrence in patients with HF hospitalized for SMVT.	There was no significant difference in all‐cause mortality between EP and SP, but EP significantly reduced VT recurrence compared with SP.
2	[16]	Spironolactone, 25 mg/day	AMI	7062	Evaluation of the benefits of routine spironolactone use after myocardial infarction/AE of SP: hyperkalemia, gynecomastia, breast tenderness	SP did not reduce the incidence of death from cardiovascular causes or new or worsening heart failure or the incidence of a composite of death from cardiovascular causes, myocardial infarction, stroke, or new or worsening heart failure.
3	[12]	Spironolactone < 50 mg	Veterans with HFpEF	52,881	Investigation of the effectiveness of spironolactone in reducing death and hospitalization outcomes for patients with HFpEF in a real‐world setting	Spironolactone use reduced all‐cause death and demonstrated a favorable trend in reducing the burden of hospitalizations.
4	[15]	Spironolactone vs Eplerenone (dose not reported)	IHD and HF	200	Comparison of the effects of SP and EP on VT recurrence in patients with ischemic heart failure hospitalized for VT episodes.	EP use significantly reduced the risk of VT recurrence compared to SP.
5	[14]	Spironolactone vs Eplerenone (dose not reported)	HFrEF	142	Determination of the efficacy of eplerenone vs. spironolactone on left ventricular systolic function, hospitalization, mortality, and clinical status/AE of SP: hyperkalemia, gynecomastia, dizziness, mastalgia‐ AE of EP: hyperkalemia, dizziness	EP had favorable effects on cardiac remodeling parameters, as well as a reduction in cardiovascular and all‐cause mortality, compared with SP in the treatment of HFrEF.
6	[20]	Spironolactone: 20 μM Eplerenone: 500 nM Canrenoic acid, a metabolite of SP: 10 nM	ACM‐DSC2‐hiPSC‐CMs	—	Assess direct effects of spironolactone and canrenoic acid ACM‐DSC2‐hiPSC‐CM to prevent ACM‐induced electrical instability.	Spironolactone and canrenoic acid corrected action potential duration, normalized K+ currents, and reduced aberrant Ca2+ events. Eplerenone was only evaluated for Ca^2+^ cycling, where it also reduced abnormal events, altogether supporting their potential therapeutic role in ACM.
7	[24]	Spironolactone, 12.5, 25, or 50 mg daily	HD	57	Evaluation of the effects of spironolactone on arrhythmic events in patients treated with maintenance HD	Spironolactone resulted in a higher frequency of bradycardia and conduction blocks compared with placebo.
8	[23]	Spironolactone, 50 mg/day	HD	30	Evaluation of the effects of spironolactone on cardiac electrical activity/AE: muscular cramps	Spironolactone increased PVCs in HD, indicating a possible proarrhythmic effect. However, improved cardiac autonomic function, as indicated by an increased HRV, may contribute to the survival benefit from spironolactone treatment in HD patients.
9	[13]	spironolactone, 25 mg/day	HFpEF	55	Evaluation of the myocardial effects of spironolactone in patients with HFpEF/AE of SP: increased serum creatinine	SP decreased the rate of accumulation of myocardial fibrosis, systolic, diastolic, and mean arterial pressure.
10	[22]	Spironolactone, 25 mg/day	ICD	90	Evaluation of the effect of spironolactone on the VT/VF in patients with ICD/AE of SP: diarrhea, gynecomastia	There was no significant difference between the median times to first VT/VF recurrence requiring ICD therapy in the two groups.
11	[18]	Spironolactone (dose not reported)	Cardiomyopathy	103	Comparison of the effect of spironolactone vs. statins and ACEi on ventricular arrhythmia rates in patients with cardiomyopathy	The average VT rate was significantly slower in patients who were actively treated with spironolactone as compared to patients on no SP, ACEi, and statins therapies, and patients treated with statins or ACEi.
12	[17]	Spironolactone (dose not reported)	LVSD and ICD	64	Evaluation of the effect of the addition of spironolactone in patients with LVSD and ICD	The addition of spironolactone as an adjunct to ICD therapy reduced monthly shocks and ICD therapies for VT.

Abbreviations: AMI, acute myocardial infarction; ACM, arrhythmogenic cardiomyopathy; ACM‐DSC2‐hiPSC CM: cardiomyocytes derived from stem cells of a patient with ACM caused by a mutation in the DSC2 gene; CMR, cardiovascular magnetic resonance; COC, combined oral contraceptive pill; DM, diabetes mellitus; EP, Eplerenone; HFpEF, heart failure with preserve ejection fraction; HFrEF, Heart failure with reduced ejection fraction; HD, hemodialysis; ICD, implantable cardioverter defibrillator; IHD, ischemic heart disease; LVEF, left ventricular ejection fraction; LVSD, left ventricular systolic dysfunction; PVC, premature ventricular contraction; SP, spironolactone; SMVT, sustained, monomorphic VT; VT, ventricular tachycardia.

The available evidence indicates that spironolactone exerts variable electrophysiological effects across different patient populations. While it has a beneficial impact on the cardiovascular system in patients with PCOS, the potential anti‐fibrotic and anti‐arrhythmic benefits in patients who suffer from HF, cardiomyopathy, and even ACM, certain subgroups, such as those with ICDs and end‐stage renal disease, may exhibit paradoxical pro‐arrhythmic responses under specific conditions. Even in patients without apparent heart disease, careful volume status evaluation, electrolyte monitoring, and rhythm control are essential to prevent rare but serious complications such as life‐threatening arrhythmias.

## 6 Conclusion

This case report is a rare, dose‐dependent side effect of spironolactone, inducing a drug‐resistant reflex tachycardia in a normotensive patient with PCOS. In clinical settings, physicians should be aware of this adverse effect and monitor patients when prescribing this medication, particularly at higher doses, even in patients without apparent structural heart disease. If tachycardia occurs, a reduction or division in the prescribed dose may be an effective and well‐tolerated management strategy.

## Author Contributions

**Zahra Abdi:** writing – original draft. **Maryam Chenaghlou:** data curation, formal analysis. **Aysa Rezabakhsh:** supervision, writing – review and editing. **Laya Hooshmand Gharabagh:** conceptualization, writing – review and editing.

## Funding

The authors have nothing to report.

## Consent

The written informed consent was obtained from the patient for publication of this case report and any accompanying images.

## Conflicts of Interest

The authors declare no conflicts of interest.

## Data Availability

The data that support the findings of this study are available from the corresponding author upon reasonable request.
